# The therapeutic promise of disrupting the PD-1/PD-L1 immune checkpoint in cancer: unleashing the CD8 T cell mediated anti-tumor activity results in significant, unprecedented clinical efficacy in various solid tumors

**DOI:** 10.1186/s40425-015-0059-z

**Published:** 2015-04-21

**Authors:** Emanuela Romano, Pedro Romero

**Affiliations:** Service of Medical Oncology, Lausanne, Switzerland; Laboratory of tumor immunobiology, Lausanne, Switzerland; Department of Oncology, Ludwig Cancer Research Center, University Hospital of Lausanne, Lausanne, Switzerland

**Keywords:** Immunotherapy, PD-1, Checkpoint blockade, Cancer

## Abstract

The role of immune checkpoints in modulating the magnitude as well as the functional profile of T cell responses is increasingly understood in molecular detail. Antibody-mediated blockade of co-inhibitory receptors has been shown to restore T cell function in both chronic viral infections and cancer. The latter has been successfully translated to new therapeutic options in the treatment of cancer. Indeed, monoclonal antibodies blocking either CTLA-4 or PD-1 have recently been approved for the treatment of metastatic melanoma in the United States, Europe and Japan. In this commentary, we summarize and put into perspective five letters recently published back to back in the November 27 (2014) issue of Nature reporting on different immunological and clinical aspects of blockade of the PD-1/PD-L1 pathway in tumor bearing hosts. Notably, treatment with anti-PD-L1 blocking antibody was shown result in profound clinical responses in patients with several solid tumor including bladder, lung and head and neck carcinomas among others. These five simultaneous publications highlight the tremendous therapeutic potential of targeting the PD-1/PD-L1 immune checkpoint and emphasize the need to identify appropriate biomarkers to guide their optimal clinical application.

## Background

Among the many strategies that cancer cells deploy to hijack the host immune defenses, blocking of the so-called immune checkpoints represents one that is showing promise to trigger a successful counter attack. Several complex safeguards are intrinsically involved in keeping the immune system from overreacting to a stimulus or mistaking a component of “self” for a dangerous invader. The programmed death 1 (PD-1) pathway is one such checkpoint regulating inflammatory responses in tissues. One or both of the PD-1 ligands, PD-L1 and PD-L2, expressed on cells in the tissues, can bind to PD-1 receptors on T cells and inhibit their function. Blocking the interaction between PD-1 and its ligands can result in enhancement of T cell activation and effector functions resulting in a swift tissue inflammatory response [1].

## Main text

Five letters recently published back to back in the same issue of Nature (November 27^th^ 2014) provide key experimental evidence on the emerging and critical role of immune therapy and more specifically, the role of immunotherapeutic antibodies that can block PD-1/PD-L1 interactions, in the treatment of cancer. In addition, they illustrate how technical progress increases the pace of identifying novel immunogenic somatic mutations as targets for the treatment of metastatic cancer [2-6]. The study by Herbst *et al*. was designed to evaluate the single-agent safety, activity and associated biomarkers of PD-L1 inhibition using the MPDL3280A, a humanized monoclonal anti-PD-L1 antibody administered by intravenous infusion every 3 weeks (q3w) to patients with locally advanced or metastatic solid tumors or haematological malignancies [2]. Across multiple cancer types, responses as per RECIST v1.1 (Response Evaluation Criteria in Solid Tumors, version 1.1) were observed in patients with tumors expressing relatively high levels of PD-L1, particularly when PD-L1 was expressed by tumor-infiltrating immune cells. Specimens were scored as IHC 0, 1, 2, or 3 if <1%, ≥1% but <5%, ≥5% but <10%, or ≥ 10% of cells per area were PD-L1 positive, respectively. In the 175 efficacy-evaluable patients, confirmed objective responses were observed in 32 of 175 (18%), 11 of 53 (21%), 11 of 43 (26%), 7 of 56 (13%) and 3 of 23 (13%) of patients with all tumor types, non-small cell lung cancer (NSCLC), melanoma, renal cell carcinoma and other tumors (including colorectal cancer, gastric cancer, and head and neck squamous cell carcinoma). Interestingly, a striking correlation of response to MPDL3280A treatment and tumor-infiltrating immune cell PD-L1 expression was observed. In summary, 83% of NSCLC patients with a tumor-infiltrating immune cell IHC score of 3 responded to treatment, whereas 43% of those with IHC 2 only achieved disease stabilization. In contrast, most progressing patients showed a lack of PD-L1 upregulation by either tumor cells or tumor-infiltrating immune cells. We would then speculate that immune therapies targeting the PD-L1/PD-1 pathway might be especially effective in tumors where this immune suppressive axis is operational, and that reversing the balance towards an immune protective environment would rekindle and strengthen a pre-existing anti-tumor immune response. Conversely, the observation of unhindered progression in those patients, whose tumors lacked PD-L1 expression in the infiltrating immune cells suggests that PD-L1 blockade either fails to promote the induction of de novo anti-tumor CD8 T cell immunity or the induced anti-tumor T cells are prevented from efficiently infiltrating the tumors. Other scenarios are of course possible. For instance, the dominance of strongly suppressive factors in the tumor milieu that potently inhibits local release of IFN-g thus failing to induce PD-L1 expression in the tumor milieu. Several candidates include TGF-b, IDO, iNOS the latter leading to the generation metabolic mediators of T cell dysfunction.

A companion study of MPDL3280A validates and extends upon those findings highlighting the relevance of the PD-1–PD-L1 pathway for the treatment of metastatic urothelial bladder cancer (UBC), a disease with unsatisfactory treatment options and dismal prognosis [3]. MPDL3280A had noteworthy activity in metastatic UBC, with responses often rapid and many occurring at the time of the first response assessment (6 weeks). This study also confirmed that tumors expressing PD-L1-positive tumor-infiltrating immune cells had particularly high response rates. A response rate of 43% (95% CI: 26–63%) achieved in advanced UBC patients with PD-L1 IHC 2/3 tumors provides evidence of noteworthy clinical activity of MPDL3280A in this setting and compares favorably with that previously seen with single-agent salvage regimens [7-10]. In addition, patients with PD-L1 IHC 0/1 tumors had a response rate of only 11% (95% CI: 4–26%). Of note, responses reported in both studies were also rapid and occurred even in patients with poor prognostic features. The safety results with MPDL3280A are encouraging as most patients did not require additional intervention– especially in patients with UBC, who are often older and have a higher incidence of renal impairment suggesting MPDL3280A may be a better option compared to chemotherapy in this patient population. Clearly, these data show that MPDL3280A is most effective in patients in whom the PD-L1/PD-1 pathway is operational to dampen pre-existing immunity. The rather unusual rapid clinical response suggests the reactivation of tumor reactive effector T cells upon antibody treatment. If so, this suggests the interesting possibility that tumor infiltrating T cells are inhibited but retain enough functional potential to eliminate efficiently tumors upon release from PD-1 mediated inhibition. Both phase I studies followed an adaptive design to allow for tumor-specific and biomarker (PD-L1 positive) enriched cohorts. An immediate benefit of this proof-of-concept trial design is that precious information can be collected more efficiently on the drug, its dosage, and on potential biomarkers of response. From an ethical perspective, this innovative design will spare more patients from receiving doses that are predictably ineffective or in irrelevant settings, and thereby ultimately allow for a more rapid assignment of alternative and potentially life-saving medicines.

Therapies that target PD-1 receptor have also shown unprecedented rates of durable clinical responses across several advanced solid cancers [11-13]. Using quantitative imaging technologies and next-generation sequencing for T cell receptors (TCRs), Tumeh and colleagues, analysed samples from 46 patients with metastatic melanoma obtained before and during anti-PD-1 therapy (pembrolizumab) and showed that responding patients had increased numbers of proliferating CD8 T cells [4]. Strikingly, they observed that pre-treatment samples from responding patients showed higher numbers of CD8 T cells with a clonal TCR repertoire in close proximity with PD-1- and PD-L1-expressing cells at the invasive tumor margin and within the tumor itself. With the aim to define reliable biomarkers of response to PD-1 blockade, the authors, using multivariate analysis, built a model based on CD8 T cell density at the invasive margin and validated it in an independent cohort of 15 patients. This further established the relevance of a pre-existing density of CD8^+^ T cells located at the invasive tumor margins as a biomarker of response in this patient cohort. Given the profound clinical implications of such observations, their prospective validation in a randomized, controlled clinical trial is warranted. In this regard, help is underway from other quarters. Indeed, in an effort to promote the immune classification of cancer as part of the routine diagnostic and prognostic assessment of tumors, a worldwide task force was initiated and the *Immunoscore Project* was launched in 2012 (http://www.sitcancer.org/about-sitc/initiatives/immunoscore). Altogether these data extend upon similar findings in the response to PD-L1 immunomodulatory antibodies and highlight that successful outcome relies upon a common mechanistic activity, whereby adaptive PD-1/PD-L1 upregulation thwarts a pre-existing CD8-mediated immune response that can be successfully rescued by blocking this immune inhibitory axis. It is however intriguing that the ensuing reactivated CD8 T cell responses be long lived. From an immunological stand point it is tempting to speculate that part of the reactivated CD8 T cells are of the memory lineage rather than purely effector T cells, as it has recently been suggested for a novel understanding of CD8 T cell exhaustion in chronic viral infection and tumors [14].

In addition to classic tumor-associated tumor antigens, numerous malignant tumors bear the potential of increased immunogenicity because of their high number of somatic mutations, depicting a mutational landscape extremely variable at the inter- and intra-patient level [15-17]. Most tumor mutations are point mutations in genes encoding intracellular proteins. Short peptide fragments derived from these *neo-proteins*, via intracellular processing and presentation at the cell surface as major histocompatibility complex class I molecules (MHCI) ligands, can elicit adaptive T cell responses. It is reasonable to expect that mutated antigen-specific T cells bear high affinity antigen receptors since they would not have been subjected to central tolerance. Until recently, there were no practical approaches to exploit the mutanome from tumors. The use of molecular cloning or biochemical approaches provided a trickle of immunogenic mutations. However, the development of several algorithms that can predict which peptides will bind to given MHC molecules are now powerful enough to establish mutant antigen discovery pipelines [18-22]. Yadav and colleagues sought to simplify the discovery of such immunogenic mutant peptides by characterizing their properties [5]. Adopting two mouse models (MC-38 and TRAMP-C1), they combined whole-exome and transcriptome sequencing analyses with mass spectrometry to identify neo-epitopes. Of the more than 1,300 amino acid changes identified, about 13% were predicted to bind MHC Class 1, a small fraction of which was validated by mass spectrometry. Using dedicated algorithms, the peptides were structurally modelled bound to MHCI. Vaccination of mice confirmed each predicted immunogenic peptide *in vivo* yielding therapeutically active T-cell responses. These compelling findings highlight that tumor mutations are useful reservoirs of exploitable neo-antigens. Using a similar approach, Castle *et al*. analyzed the mutanome of the widely used B16 melanoma cell line and tested 50 MHC-binding m-peptides, 16 of which were immunogenic and 11 of which preferentially recognized the mutant peptide over the wild-type counterpart. Importantly, they showed that vaccination with 2 of those suppressed the growth of established B16 melanomas [23].

In a time of intense quest for personalized modalities for cancer therapy, immune interventions that aim at priming or boosting anti-tumor immune responses tailored to mutational heterogeneity holds much promise. Consistent with this concept, Gubin et al. employed genomics and bioinformatics approaches to identify tumor-specific mutant proteins as a class of T-cell rejection antigens following anti-PD-1 and/or anti-CTLA-4 therapy. They demonstrate that in mice bearing aggressive sarcomas therapeutic synthetic long-peptide vaccines incorporating these mutant epitopes induced tumor rejection comparably to checkpoint blockade immunotherapy [6]. One potential caveat of this study, as is true in similar tumor models, is to what extent chemically-induced, highly immunogenic murine tumors reproduce the biology of human cancers. We speculate that the TCR repertoire in responding patients should be largely overlapping and show oligoclonal expansion, and that mutational load should correspond to signs of CD8 T cell proliferation and activation. This has been elegantly shown in a patient with advanced melanoma responding to ipilimumab therapy, where cancer exome-guided analysis of T-cell reactivity revealed a specific response against two neoantigens, whose magnitude increased significantly upon therapy [18]. This has also been observed in a series of melanoma patients, where somatic neoepitopes that elicited an antitumor response were augmented by and associated to clinical response to CTLA-4 blockade [24].

## Conclusions

Blockade of the immune-inhibitory PD-L1–PD-1 pathway has shown remarkable efficacy in patients with advanced NSCLC, melanoma, renal-cell cancer, and Hodgkin’s lymphoma including upon failure to several lines of therapy [13,25-27]. According to the recent literature, blockade seemed particularly effective in subjects with pre-existing cellular immune response [7-10]. Upregulation of the PD-1–PD-L1 signaling axis in tumor tissue, as a consequence of type I IFN activation and invasion by T cells, predicts therapeutic benefit from PD-L1–PD-1 blockade alone. PD-L1 expression – particularly, by the tumor-infiltrating immune cells warrants prospective validation as a potential biomarker in clinical trials investigating PD-1–PD-L1 antibody-containing regimens. However, the need of a CLIA-based standardized assay for PD-L1 expression and the substantial complexity of the tumor–host cross-talk should be taken into account as they might influence outcome with respect to any single or combined intervention.

The recent results have clear and immediate clinical effects: anti–PD-1–PD-L1 therapy will become the foundation of the oncology panoply, possibly sparing patients toxic effects of chemotherapy, where deep investigation into tumor mutational load and immune interventions could usher a new wave of promising therapies. We predict that the list of cancers sensitive to such therapeutic intervention will also extend to other cancers, including colorectal adenocarcinoma (especially that harbouring microsatellite instability) and other gastrointestinal cancers, and mesothelioma. As demonstrated in the setting of Hodgkin’s lymphoma, the mechanism(s) of response to anti–PD-1 therapy need(s) further study. Although Hodgkin’s lymphoma is characterized by genetically driven PD-L1 and PD-L2 overexpression and an inflammatory response, evidence for a tumor antigen-specific response has not yet been described.

With recent data showing impressive clinical activity of PD-1 or PD-L1 antagonists in subgroups of patients with a variety of different cancers, the critical and foundational role of immune interventions in cancer treatment is becoming a reality and treatment indications are becoming more clearly defined. However, a subset of cancers are poorly infiltrated with immune cells (i.e. at least one third of all metastatic melanomas) [28] and, consistent with recently published work patients bearing tumors, in which type I IFN–regulated genes, T cell–related genes, and PD-L1 are expressed at low levels, have comparatively poor prognosis [29,30]. Thus, understanding the profile of this patient subgroup will most likely pay off, possibly revealing the diversity of mechanisms controlling antitumor immunity and suggesting new strategies to promote cancer immunity and clinical benefit. Immune therapies promoting CD8 T cell infiltration of tumors should help in patients not responding or relapsing on these treatments. Our expectation is that, in this catergory of patients amongst the modalities to augment tumor immunogenicity, the optimization of vaccines and oncolytic viruses in synergistic combination with checkpoint blockers or costimulatory receptor agonists are promising. Chemotherapeutic and targeted agents, as well as radiation therapy are also being exploited in this context, as data have shown that each of those approaches can induce immunogenic cell death, intra-tumoral infiltration of immune cells, and increase in antigen presentation, respectively. Moreover, the combination of checkpoint inhibitors or costimulatory receptor agonists with cytokines such as IL-2, IL-15 or type I interferons could be tested in patients not responding or relapsing on these treatments. Finally, as the tumor hijacks vascularization during its natural history, one potential synergism to augment clinical benefit could be the combination of immunotherapy with biologic agents to normalize tumor vasculature allowing better immune cell influx to the tumor. These recent years of immunotherapy success presage the substantial additional gains that could be achieved from continued research endeavours in this field and substantiate the dedication of the so many investigators in the game.
